# Clinical validation of a novel hand dexterity measurement device

**DOI:** 10.1371/journal.pdig.0000744

**Published:** 2025-03-10

**Authors:** Conor D. Hayden, Bruce P. Murphy, Deirdre Gilsenan, Bahman Nasseroleslami, Orla Hardiman, Deirdre Murray

**Affiliations:** 1 Trinity Centre for Biomedical Engineering, Trinity Biomedical Sciences Institute, Trinity College Dublin, Dublin, Ireland; 2 Department of Mechanical, Manufacturing and Biomedical Engineering, School of Engineering, Trinity College Dublin, Dublin, Ireland; 3 Academic Unit of Neurology, Trinity Biomedical Sciences Institute, Trinity College Dublin, Dublin, Ireland; 4 Advanced Materials and Bioengineering Research Centre (AMBER), Trinity College Dublin, Dublin, Ireland; 5 Neurocent Directorate, Beaumont Hospital, Beaumont, Dublin, Ireland; Khulna University, BANGLADESH

## Abstract

The lack of sensitive objective outcome measures for hand dexterity is a barrier for clinical assessment of neurological conditions and has negatively affected clinical trials. Here, we clinically validate a new method for measuring hand dexterity, a novel hand worn sensor that digitises the Finger Tapping Test. The device was assessed in a cohort of 180 healthy controls and 51 people with Amyotrophic Lateral Sclerosis (ALS) and compared against rating scales and traditional measures (Nine Hole Peg test and grip dynamometry). 14 features were extracted from the device and using a logistic regression algorithm, a 0-100 dexterity performance score was generated for each participant, which accounted for age/sex differences. The device returned objective ratings of a participant’s hand dexterity (dominant, non-dominant and overall score). The average overall dexterity performance score in all healthy participants was 88 ± 17 (mean ± standard deviation). The overall dexterity score was statistically significantly worse in participants with ALS (age/sex matched healthy subset: 80 ± 20, ALS: 45 ± 32, p-value < 0.0001). The device also had a higher completion rate, (94% dominant hand) compared to the traditional measures (82% dominant hand). This test and scoring system have been validated and the regression model was developed using a framework that is potentially applicable to any relevant condition. This device could act as an objective outcome measure in clinical trials and may be useful in improving patient care.

## Author summary

Clinical hand dexterity assessment methods have remained static for decades. Issues including subjectivity, floor effects and clinical accessibility have not been conclusively resolved. We have developed a novel hand worn sensor that digitises a common neurological test, the Finger Tapping Test. In this study we present the results of a large validation study (180 healthy controls and 51 people with Amyotrophic Lateral Sclerosis) which has provided a test and scoring system for hand dexterity measurement that returns an objective dexterity performance score. Unlike the current gold standard rating scales, it is not affected by hand dominance. Our device accounts for age/sex differences and has a higher completion rate than the other traditional tests. The device presented here has the potential to act as an objective outcome measure in clinical trials. We believe it has significant advantages over the current methods which were developed decades ago. Hope for improvements in treatments for neurodegenerative conditions relies on sensitive detection of meaningful change in function in clinical trials of new therapies. Drug companies and clinical trial providers will only adopt proven, sensitive digital devices and the device described provides an opportunity for such change.

## Introduction

Impairment in hand dexterity is a feature of many neurological diseases such as Parkinson’s Disease [1], Amyotrophic Lateral Sclerosis (ALS) [2], Stroke [3] or Multiple Sclerosis [4]. Loss of hand dexterity has a significant impact on independent functioning in daily life. Sensitive, reliable and low burden tools to assess and monitor hand impairment are important for diagnosis and to accurately evaluate the outcome of interventions. Currently, subjective rating scales are widely used [5–7], but are vulnerable to rater bias, cannot differentiate in the dominant versus non-dominant hand [8], lack sensitivity, display floor effects, non-linear decline and low reliability and are limited to being recorded at episodic clinic visits [9]. The psychometric limitations of rating scales, be they single item or multiple item, have been well established [10]. The scales lack conceptual clarity [11], with items assessing different concepts or lacking the ability to capture the variable nature of the disease [12]. Despite these concerns rating scales are still used as the primary or secondary outcome measure in many clinical trials [13–15], potentially missing clinically meaningful improvements or changes in rates of decline in outcome post experimental medical interventions.

Performance-based tests such as the nine-hole peg test (NHPT) [16], Jebsen Hand Function test [17], Perdue Peg Board test [18] and grip strength tests [19] partially address the limitations of subjective rating scales. However, each of these tests also have issues with floor and/or ceiling effects, significant performance differences between the dominant and non-dominant hands and/ or between the sexes, poor reliability and in some cases accessibility in the clinical setting [20].

**Fig 1:**
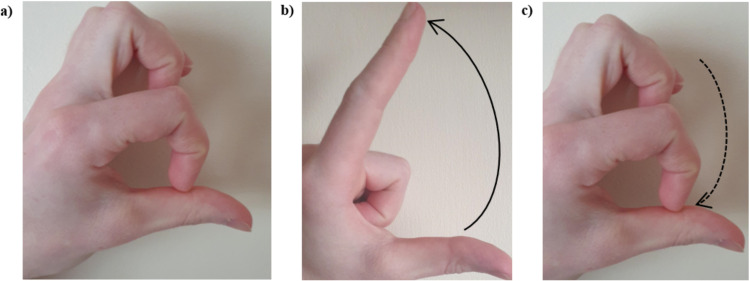
Finger tapping test (FTT) sequence with and without the dexterity device. The person being assessed begins with the tip of their index finger at the distal crease of their thumb (A), they then raise the index finger as high as possible (B) and then return to the start position (C). This is repeated a number of times and as quickly as possible.

The Finger Tapping Test (FTT) (Fig 1a–1c) is a quasi-objective assessment, which is quick, easy and widely used in clinical practice [21]. The rater visually evaluates the size, speed and accuracy of a finger tapping movement, noting decrement with repeated movement. It is subjectively scored as normal/abnormal or using a Likert scale [22,23]. However, scoring is subjective and objective information such as speed, height and accuracy of the movement are lost, limiting tracking of longitudinal change.

Technology has the potential to provide objective, sensitive measurement through digitisation of existing tests such as the FTT. Potential technology-based solutions range from using accelerometers [24], gyroscopes [25], magnetometers [26], glove-based devices [27], camera-based solutions [28,29], virtual environment devices [30], smartphones [31] and tablets [32]. However, none of the currently proposed solutions have been widely adopted into clinical practice, due to limitations in usability, lack of validation, cost and clinical meaningfulness [20].

In summary, there is no ideal measurement of hand dexterity, which capitalises upon modern technologies currently available within a clinical setting and accounts for hand dominance and sex differences. Here, we present the clinical validation of a novel hand dexterity assessment device along with a scoring framework, accounting for age and sex differences which provides a dexterity performance score.

## Results

### Participant demographics and results of traditional measures

Participant characteristics for the 180 healthy participants and 51 people living with ALS (PALS) are presented in Table 1.

**Table 1:** Participant characteristics.

Participants (n=231)	Healthy (n=180)	PALS (n=51)
**Male sex** (no. (%))	90 (50%)	32 (63%)
**Age (years)** Mean ± SD	50 ± 17	60 ± 10
**Dominant Hand (no.)** Right	160 (89%)	50 (98%)
**Rating Scales (Mean ± SD)**		
ALSFRS-r/48	NA	36 ± 9 (n = 50)^*^
ALSFRS-r UL Subscore/12	NA	8 ± 4 (n=51)^*^
DASH/100	NA	39 ± 29 (n=49)^*^

PALS: People with ALS, ALSFRS-r: ALS Functional Rating Scale revised questionnaire, DASH: Disability of Arm, Shoulder and Hand questionnaire; no.: number; SD: standard deviation.

* missing data: One ALSFRS-r and two Disability of Arm, Shoulder and Hand questionnaires (DASH) were not completed.

The results of the traditional tests for the 180 healthy participants, by sex are presented in S1 Table. The test completion rate for the traditional measures was 100% for the healthy participants. For PALS, lower completion rates were achieved; NHPT (82%, 74%), grip strength (82%, 80%), tip pinch (86%, 84%), palmar pinch (86%, 80%) and key pinch (92%, 90%) (dominant and non-dominant hands respectively).

Correlation matrices between the grip strength test and pinch gauge tests are shown in S2 Table. Moderate to strong correlations were seen between the grip strength test and the pinch gauge tests as well as within the three pinch gauge tests. Therefore, only one strength test, the grip strength test, was utilised for further comparisons.

**Fig 2:**
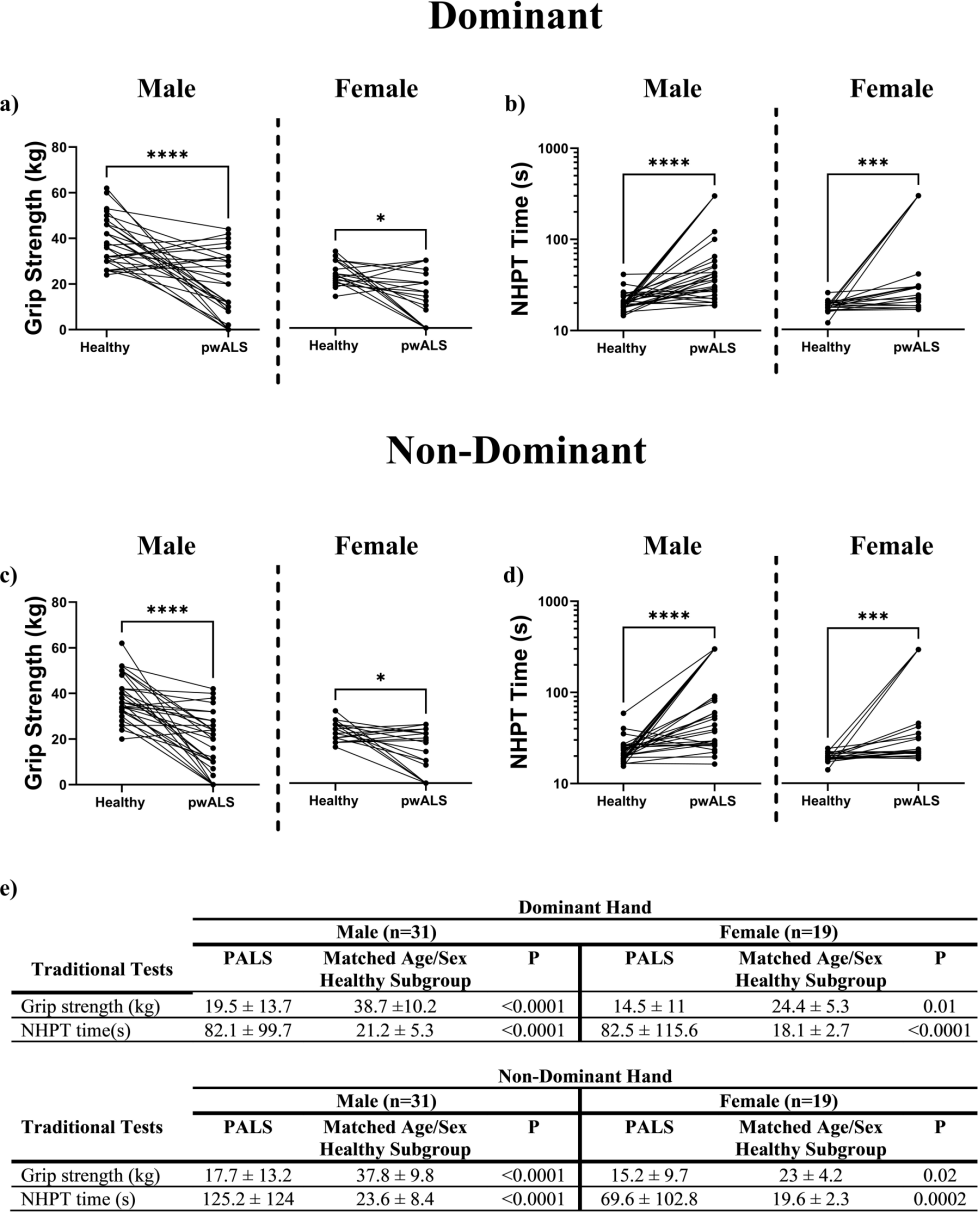
Results from the performance-based assessments of hand function for PALS and the age/sex matched healthy subgroup. a-d show the results (mean and standard deviation) for the dominant and non-dominant hand separate by sex. Wilcoxon matched pairs signed rank tests were carries out between each group. Significance is denoted by (*) using the convention **p** < 0.05 (*), **p** < 0.01 (**) and **p** < 0.001 (***) or ns when no significance is noted.

Results of traditional upper limb function tests for 50 PALS (traditional tests not completed by one male), and a sub-set of 50 healthy participants age and sex matched to the PALS are presented in Fig 2. The results of the traditional tests illustrate that hand dexterity/function is significantly worse in the PALS, in all measures, compared with the healthy participants. Also, as significant differences were observed in the dominant and non-dominant hands for both sexes, only the dominant hand results are shown for some of the subsequent analysis, with the non-dominant hand results in the accompany supplementary material (S2 Fig).

### Dexterity device results in healthy participants and comparison with traditional measures

The dexterity device returned 14 distinct features from the FTT procedure, with S3 and S4 Tables 4 showing the results in the healthy participant’s dominant hand and non-dominant hands in three age categories (20-39, 40-59, 60+), separated by sex. These features were processed to output dexterity performance scores representing performance of the dominant and the non-dominant hands, which were statistically significant across the three age categories (S3 and S4 Tables).

The average calculated dexterity performance score in all healthy participants (n=180) was 88 ± 19/ 87 ± 18 (mean ± standard deviation) in the dominant and non-dominant hands respectively. The individual hand scores were then combined to provide an overall dexterity score of 88 ± 17, which reflected function in both hands. The test completion rate for the dexterity device was 100% for the healthy participants.

**Fig 3:**
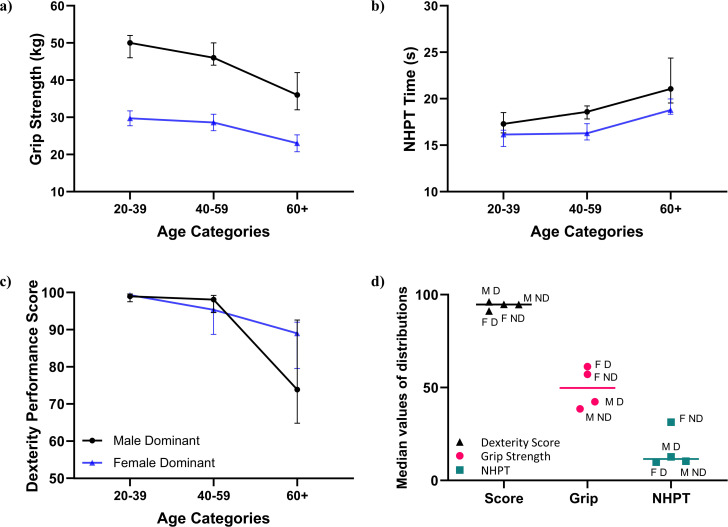
Results for the dominant hand across three age categories for healthy participants, by sex. a The grip strength test. b The nine hole peg test (NHPT). c The dexterity performance score (from 50 units). d The normalised median values of the distributions calculated from min-max scaling transforms plotted for the three tests using the abbreviations **M:**Male, **F:** Female, **D:**Dominant and ND: Non-Dominant. For (a-c) the median and 95% confidence intervals are plotted and separated by sex and hand dominance while (d) plotted the normalised median values around the overall median value for that test.

The difference in performance with age for the dexterity device along with the two traditional tests of interest (grip strength and NHPT), separated by sex is illustrated in Fig 3a–3c) for the dominant hand (see S1 Fig for non-dominant hand results). There are no differences seen among the healthy participants in the dexterity performance score with sex or hand dominance, unlike in the traditional tests. This is demonstrated in Fig 3(d) when the normalised median values of the distribution of the grip strength, NHPT and score, separated by sex are plotted.

### Dexterity device results in PALS and comparison with age/sex matched healthy cohort

The majority of the 14 features from the FTT procedure were statistically significantly lower in the PALS compared with the age and sex matched healthy cohort for the dominant and non-dominant hands (Table 2). The same features, separated by sex are reported for the dominant and non-dominant hands in S5 and S6 Tables respectively. The dexterity performance scores were similarly statistically significantly lower in PALS compared to the subset of healthy participants (Table 2 and S4 Fig). The overall dexterity score, which reflected function in both hands, was statistically significantly worse in PALS (healthy subset: 80 ± 20, PALS: 45 ± 32, p-value <0.0001).

**Table 2:** Results of the dexterity device features and calculated dexterity score for PALS compared with age and sex matched healthy participants. The results (mean and standard deviation) are presented for the dominant and non-dominant hands. Significant tests were carried out using a paired t-test or Wilcoxon matched pair signed rank test.

	Dominant Hand			Non-Dominant Hand		
	Healthy (n=51)	PALS (n=51)	P	Healthy (n=51)	PALS (n=51)	P
Time to Completion (s)	5.9±2	12.2±10.7	^***^	6.2±1.9	11.1±7.4	^***^
Avg. Extension Height (mm)	110.3±18.2	82.6±38.5	^***^	108.8±14	79.3±32.5	^***^
Max Extension Height (mm)	117.9±17.7	90.4±39.6	^***^	115.5±13.9	85.6±32.7	^***^
Avg. Extension Passive Height Score	0.8±0.1	0.7±0.3	^***^	0.9±0.1	0.7±0.3	^***^
Max Extension Passive Height Score	0.9±0.1	0.8±0.3	^**^	0.9±0.1	0.8±0.3	^***^
Avg. Hesitation Time (s)	0.2±0.1	0.4±1.2	ns	0.1±0.1	0.2±0.7	^*^
Avg. Hesitation Height (mm)	2.7±2.4	4.2±11.8	^**^	1.6±2.1	1.4±7	^**^
Avg. No. of Hesitations per Test	6.9±2.5	6.6±11.5	^**^	4.7±3.3	3.3±7.2	^**^
Avg. No. of Hesitations per Tap	0.9±0.7	3.4±11.8	^*^	0.6±0.6	1.1±7	^***^
Avg. Accuracy (mm)	4.8±2.9	34±117.7	ns	5.2± 2.9	14.3±69.5	ns
Decrementing amplitude (%)	8.2±7.2	42.9±117.4	ns	7.7±5.7	20.6±69.5	ns
Avg. Extension Speed (m/s)	0.5±0.1	0.2±0.2	^***^	0.4± 0.1	0.2±0.2	^***^
Avg. Contraction Speed (m/s)	0.4±0.1	0.2±0.2	^***^	0.4± 0.2	0.2±0.1	^***^
Avg. Speed (m/s)	0.4±0.1	0.2±0.2	^***^	0.4±0.1	0.2±0.1	^***^
**Dexterity Score**	**80 ± 23**	**43 ± 35**	^***^	**80 ± 22**	**47 ± 35**	^***^

P: p-value; s: seconds; mm: millimetres; %: percentage; m/s: meters per second, avg: average. Significance is denoted by (^*^) using the convention p < 0.05 (^*^), p < 0.01 (**) and p < 0.001 (^***^) or ns when no significance is noted.

The dexterity performance score in PALS was moderately correlated with the traditional tests (Table 3).

**Table 3:** The correlation coefficients between the dexterity performance score, the two main traditional measures (grip strength and NHPT) and the two rating scales (DASH and ALSFRS-r) are presented for PALS. The coefficients, using Spearman’s correlation test, were calculated against the overall dexterity performance score and by dominant and non-dominant hand, where appropriate.

Features	Overall dexterity score	
DASH	-0.57	
ALSFRS-r	0.46	
ALSFRS-rUL	0.49	
	**Dominant hand dexterity score**	**Non-Dominant hand dexterity score**
Grip Strength	0.38	0.44
NHPT	-0.64	-0.64
DASH	-0.60	-0.38
ALSFRS-r	0.42	0.40
ALSFRS-rUL	0.62	0.28

**Fig 4:**
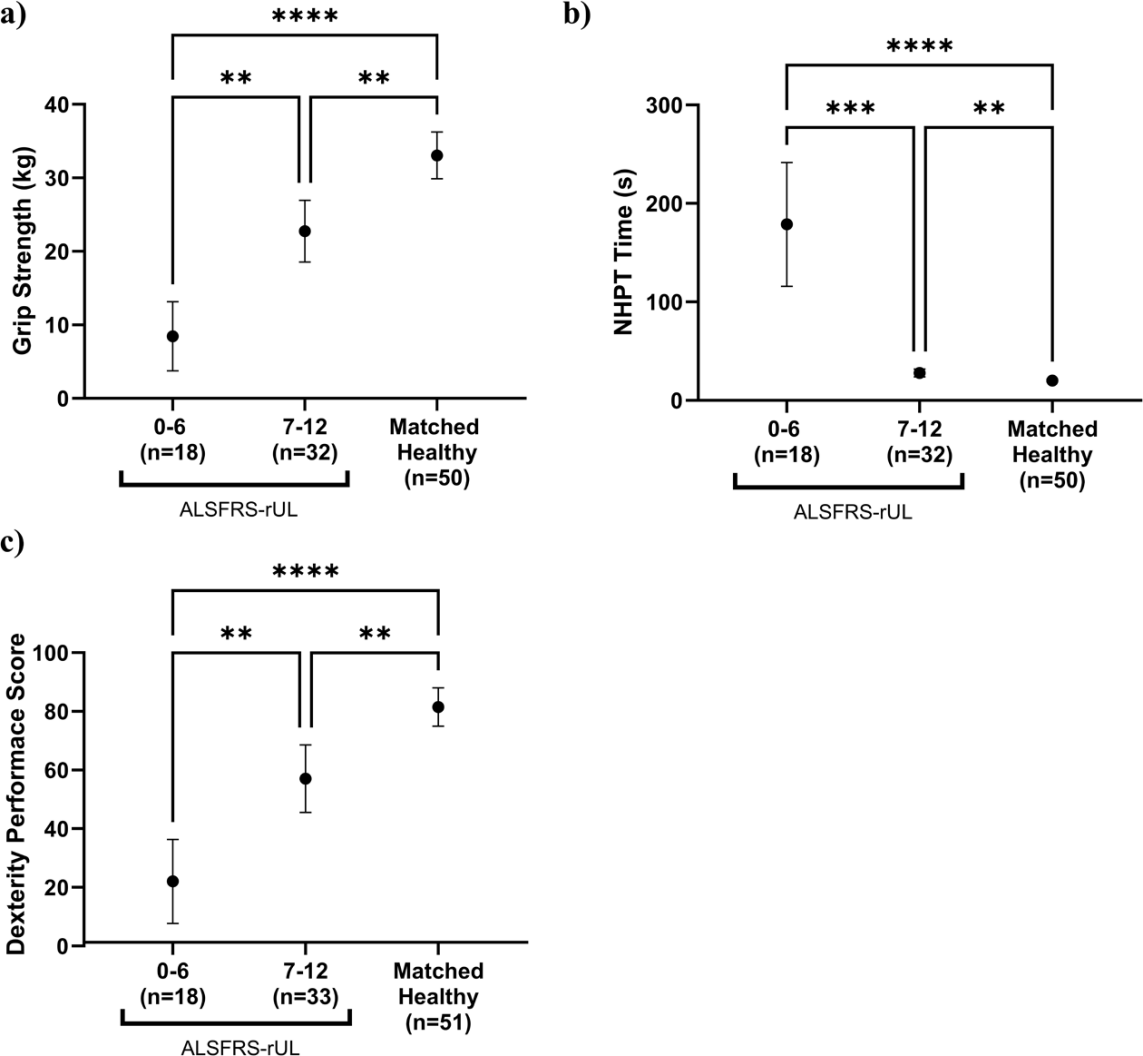
Results (mean and 95% confidence interval) for the PALS, divided into two ALSFRS-rUL groups compared against the age/sex matched healthy cohort for dominant hand. a Grip strength test. b Nine hole peg test (NHPT). c Dexterity performance score. Pairwise comparisons were carried out using a Kruskal-Wallis (with a Dunn’s multiple comparison test) test. Significance is denoted by (*) using the convention p < 0.05 (*), p < 0.01 (**), p < 0.001 (***) or p <0.0001 (****) or ns when no significance is noted.

The grip strength, NHPT and dexterity performance scores were plotted by two predefined ALSFRS-rUL scoring categories (0-6 = severely impaired upper limb function, 7-12 = less severely impaired upper limb function) and compared with the healthy subset (Fig 4(a–4c) for dominant hand and S3 Fig, Fig 3a–3c) for non-dominant hand), which demonstrates the differences in test performance.

### Dexterity device utility

A learning effect was identified with repeated testing in healthy participants; in the dominant hand 23 first tests (13%), 59 second tests (33%) and 98 third tests (54%) were the ‘best test’, with 23 first tests (13%), 82 second tests (45%) and 75 third tests (42%) ‘best tests’ in the non-dominant hand. Similarly, for PALS (after the participants who were unable to record a result were excluded), in the dominant hand 14 first tests (29%), 15 second tests (31%) and 19 third tests (40%) and in the non-dominant hand 10 first tests (20%), 19 second tests (38%) and 21 third tests (42%) were identified as the best test.

**Fig 5:**
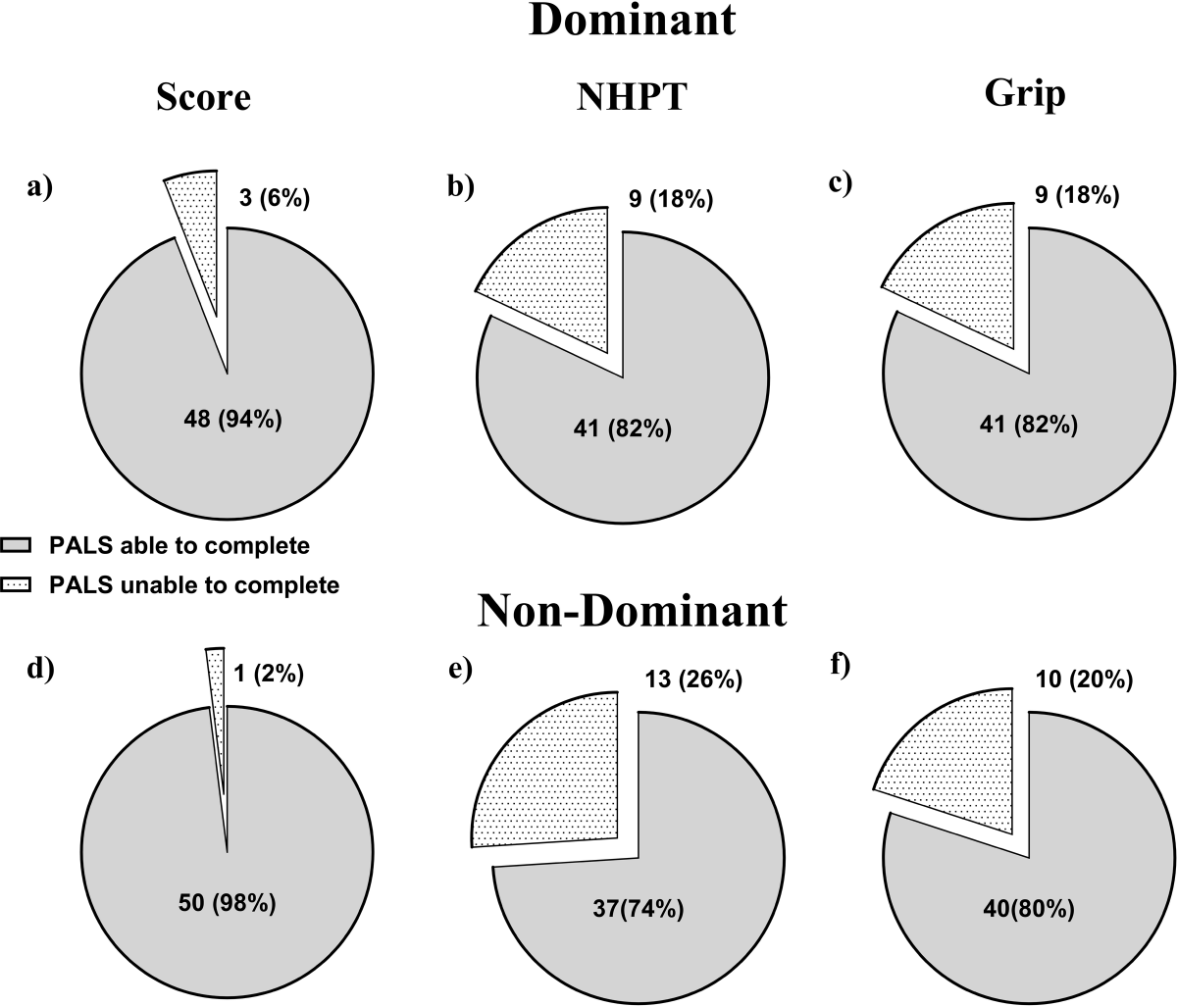
Completion percentages in PALS for the dexterity performance score and the two traditional tests of interest. a-c shows the results for the dexterity performance score, NHPT and grip strength for the dominant hand. d-f show the result in the non-dominant hand.

For PALS the completion rate of the dexterity device was 94% (three unable) in the dominant hand and 98% (one unable) in the non-dominant hand. This is greater than the completion rate for the two traditional tests (Fig 5).

The System Usability Scale (SUS) was completed by n=10 users of the dexterity device and returned a score of 86 ± 14 (mean ± standard deviation). The scoring range for the SUS is 0 - 100 with 100 the best possible score.

25 healthy participants (14 Male, average age 39 years old) were included in analysis of the dexterity device reliability. This provided Inter Class Correlation (ICC) coefficients of 0.91 and 0.85 for the dominant and non-dominant hands and also returned no significant difference between the two groups when the score was calculated.

## Discussion

Clinical hand dexterity assessment methods have remained static for decades. There is no technologically modern solution that has successfully translated from research to clinical use [20]. Limitations including subjectivity, floor effects and clinical accessibility of outcome measurement tools have not been conclusively resolved. To address this, a novel technology-based dexterity assessment device that returns an objective dexterity performance score based on quantifying a participant’s performance on the finger tapping test has been developed. This test and scoring system have been validated in a large healthy cohort and in a cohort of people affected by ALS, a neurodegenerative condition of interest. The dexterity device has a lower floor effect than traditional measures, allowing objective assessment of hand dexterity, even when it has significantly declined. The objective dexterity performance score is significantly different in the healthy cohort compared with the cohort with ALS and is significantly different in PALS at different stages of disease progression. The dexterity device has excellent test-retest reliability and a high usability score. The device has the potential to be a new and better tool to measure hand dexterity.

### Performance in healthy people

The dexterity performance score provides a rating of a participant’s hand dexterity, while accommodating age and sex-based differences in performance, i.e., if a person has good hand dexterity, they will receive a score tending towards one hundred regardless of sex (Fig 3c). The min-max transformation of the dexterity performance score, grip strength test and NHPT (Fig 3d) shows that the dexterity performance score results clustered around the median, whereas a sex effect was clearly present in the grip strength results, while the female non-dominant hand result for the NHPT had a different distribution from the other tests. These sex based differences make interpretation of these traditional tests difficult, which has been addressed by the dexterity scoring system. Lower scores within the healthy cohort were indicative of declining hand function, where a trend away from a perfect score is apparent in males and females over 60 years of age and may demonstrate age related changes in function.

The ALSFRS-rUL score (0-12) was divided into 2 scoring categories representing more (0-6) and less severely impaired (7-12) hand function. There were significant differences in the dexterity score between these ALSFRS-rUL scoring categories. This was also apparent with the traditional tests, supporting the validity of the novel measure. Furthermore, significant differences were found between the PALS and the matched healthy cohort (Fig 4).

## Advantages over traditional measures

The user experience data collected during testing indicated that assessment using the dexterity device is efficient (less than 5 minutes per test), and that the device is comfortable to use for the patient. A previous paper has The device accurately collects comprehensive data, that quantifies complex movements associated with a finger tapping test [33]. Some PALS, who had severely impaired hand function, were unable to complete some of the traditional tests. This occurred more frequently for Grip Strength and the NHPT than for the novel dexterity test, which was the most accessible test (Fig 5). An inability to complete tests resulted in allocation of default scores (0 kg for the grip strength and dexterity device and 300 seconds for the NHPT). The default scoring resulted in limitations in interpretation of results, particularly for the NHPT. The higher completion rate for the dexterity device (94 and 98% for the dominant and non-dominant hand respectively) and the defined 0-100 scoring range ensured that this issue was less problematic for interpretation of results for the dexterity device output.

The dexterity device provides a score representing function in each hand and can be further processed to provide one overall dexterity score. In contrast the ALSFRS-r and DASH cannot differentiate between different levels of functioning in each hand [8], while the Grip strength and NHPT are limited to providing scores for each hand. The limitations of the questionnaire assessments are demonstrated by the example of one participant, who had an ALSFRS-rUL score of zero, a DASH score of 76 (indicating a significant level of disability), was unable to complete the grip strength test in both hands, and only completed the NHPT using their non-dominant hand in 60 seconds. The levels of functioning in each hand was reflected in dexterity scores of 0.02 in the dominant hand and 0.13 in the non-dominant hand. Additionally, the differences in correlations between the dexterity device performance score in the dominant and non-dominant hands and the single ALSFRS-rUL, (0.62 D/0.28 ND), indicates the inability of the ALSFRS-rUL score to reflect differences between dominant and non-dominant hands. Similar differences in correlation were seen for the DASH questionnaire.

### Robustness of the model design

The machine learning model, logistic regression, used to calculate the coefficients for each variable in the model is straightforward to implement and interpret. It also adjusts for the effects of any multicollinearity in the model. The model is alert to small changes or trends in the features that would be missed by gross examination of individual features and is an efficient method of combining the individual features scores generated by the dexterity device. Time to completion, height and speed features were the primary features that differentiated the groups (S3 and S4 Tables, Table 2). However, it would be impractical to interpret these features independently when examining differences between groups. The regression model included all 14 features extracted from the digitised FTT, which means that there was no information loss. Any changes from healthy performance in individual features contribute to the performance score based on their relative importance. By using the Leave-One-Out Cross Validation method to generate the dexterity performance scores for each participant, the bias in the model design was reduced. The score for each participant was calculated using the coefficients from a model that did not contain that participant.

## Dexterity device learning effect

There was a learning effect associated with the device. The first test was chosen for analysis in only 13% of the healthy participant tests for both hands and while it was less pronounced in the PALS tests, it was clear that at least three tests should be completed by participants (if able) for both hands. The device’s learning effect was in line with other assessments such as the NHPT. which is recommended be administered five times (four times pre-baseline) [34]. The effect of fatigue must be considered in a patient population and the increased demand with repeated testing may be challenging and reduce the appeal of using the device.

## Limitations and future work

The current testing procedure for the device used a hand brace to standardise the movement for all participants. This also allowed participants to complete the test in a gravity neutral position ensuring that participants with weaker hand function were able to reach their maximum height. This may limit application in the clinical setting. There was an implicit assumption in the logistic regression model design, that because PALS are diagnosed with a condition that affects hand function, the difference between the features for that cohort and a healthy one could be used to determine the coefficients for the model. For the device and dexterity performance score to be reliably used on people diagnosed with any other neurological condition (for example Parkinson’s Disease), the model must be re-tested and validated on that other cohort, to assess the difference, if any, this would make to the coefficients used. Sensitivity to change was not fully addressed in this study. A longitudinal study is necessary and is in process to quantify what level of change is meaningful in the dexterity performance score.

## Conclusion

In this paper, we have demonstrated that the novel dexterity device is a better way of measuring hand dexterity compared to the traditional measures. Unlike traditional measures the novel device provides a single dexterity performance score for all participants. It also accounts for sex and age effects. If implemented in the clinic in the future, it may enhance the objective measurement of hand dexterity.

## Materials and methods

### Participants

This study included two cohorts: healthy people and people living with Motor Neurone Disease/Amyotrophic Lateral Sclerosis (PALS), a neurodegenerative condition commonly affecting hand function. Ethical approval was granted by the appropriate research ethics committees, Trinity College Dublin’s School of Medicine for testing in healthy participants (REC 20190209) and Beaumont Hospital Dublin for PALS (REC 18/25). Potential participants were invited to participate by the researchers in person: who explained the study, provided an information leaflet and answered any questions, after which written consent was obtained. The raters were not blinded to the participant health status. Participants attended one testing session which took approximately 25 minutes. Demographics, including sex, age, hand status and diagnosis (if required) were recorded along with testing time and any participant discomfort.

The healthy cohort included 180 participants comprising 30 males and 30 females in each of three age categories (20-39, 40-59, 60+). For inclusion, participants were required to be healthy and able to give informed consent. Participants were excluded if they had a diagnosis of a neurological condition or any condition affecting hand function. 51 PALS were recruited. For inclusion, participants were required to be able to give informed consent and had to be diagnosed with ALS. Participants were excluded if they had complete paralysis of the arm which meant that they were unable to complete any of the tests.

## Traditional measures

All participants were tested using three traditional assessment tools: (i) grip strength dynamometry, (ii) pinch gauge (tip pinch, palmar pinch and key pinch) and (iii) the Nine Hole Peg Test (NHPT), while PALS additionally completed two questionnaires: (i) the Disability of Arm, Shoulder and Hand (DASH) and (ii) the ALS Functional Rating Scale Revised (ALSFRS-r). The DASH is a 30-item self-reported questionnaire that asks the participant to rate their ability to complete functional tasks. It’s graded on a 5-point (0-4) Likert scale with a score of 0 indicating normal function and a score of 100 indicating no function. The ALSFRS-r is a 12-item scale with a maximum score of 48, which is comprised of 4 subscales (bulbar, upper limb function lower limb function and respiratory function) each scored out of 12. Only the three upper limb (UL) function questions (Q4, 5 and 6) are relevant when assessing the dexterity device denoted by (ALSFRS-r UL). Each question is rated from 0-4 with 4 indicating normal function.

The traditional tests were conducted as per their standard operating procedures [35–37]. Three trials of each test were completed on each hand (if able) with the exception of the NHPT, where two tests were completed. Participants who were unable to complete the grip dynamometry or the pinch gauge tests were given a score of 0 kg while those who were unable to complete the NHPT were given a score of 300 seconds. The best value for each test (highest force for the grip/pinch gauge tests and quickest time for the NHPT) was used for analysis.

## Dexterity device

**Fig 6:**
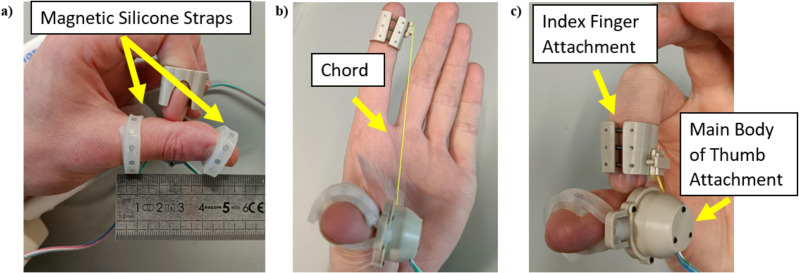
Photographs of the system in situ on a person’s hand. a and c show the thumb and finger attachment components in a contracted state. b shows the device is shown in an extended configuration. The movement of the chord is captured by the device which inherently captures the movement of the finger relative to the thumb.

The dexterity device (Fig 6) used in this study was designed by our group and is a simple to use mechanical device with an integrated app [33]. This device captures the distance a participant’s index finger extends from the distal crease of the participant’s thumb, with respect to time (Fig 2a–2c). This device can record the repetitive movements of the tip of the index finger to/from the distal crease of the thumb. The device has been shown to be comfortable, quick to use, repeatable and accurately collects comprehensive data, that quantifies movements associated with a FTT.

**Fig 7:**
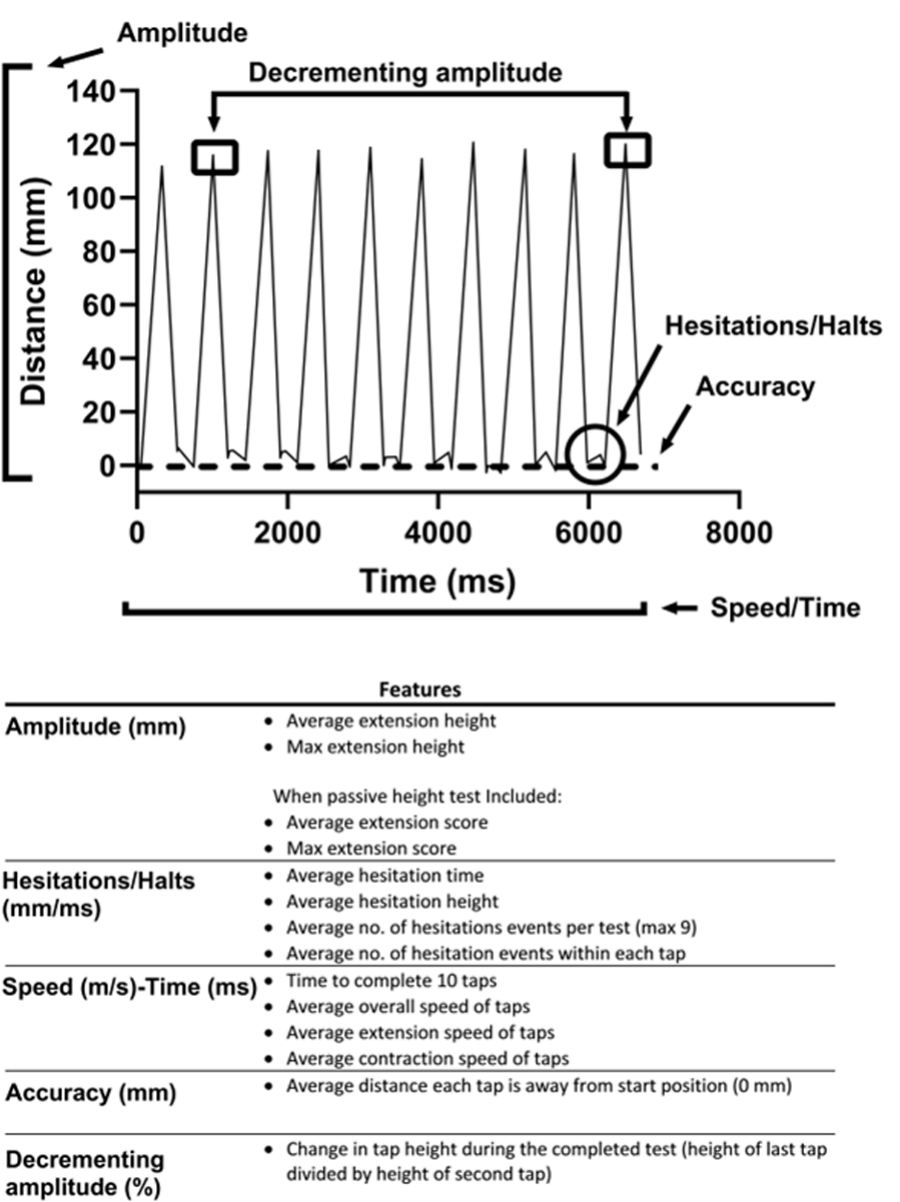
Graph of a participant’s raw data showing the features extracted from a completed FTT.

The test method for the dexterity device was standard for all participants. Firstly, they rested their hand in a custom hand support to standardise orientation and ensure the movement was carried out in a gravity neutral position (S4 Fig). The tester attached the device to their thumb and index finger. Once the device was secured, they were instructed to place the tip of their index finger on the distal crease of their thumb and to extend their finger as high and as fast as they could, returning to the starting point of the distal crease between each tap, for eleven taps, ten of which were used for analysis. The two outputs from the test were distance travelled from the distal crease of the thumb and the corresponding time. Participants first completed a number of practice taps to ensure that the device was correctly secured. Three tests for both hands were completed. A ‘best test’ was chosen by isolating the FTT that met the criteria of shortest time and highest average extension height. A separate test, to record the maximal passive height of the movement was carried out once for each hand, which involved the tester passively extending the participant’s index finger to the maximum potential height. Fig 7 shows the 12 features that are extracted from a participant’s raw data graph. An additional two features derived from the passive height test are also calculated. These features are the average and maximum height scores which return an output between 0-100, when the average and maximum extension height values are divided by the corresponding passive height value for a participant’s hand.

Following testing, participants were asked to provide feedback regarding comfort using the device. The test-retest reliability of the dexterity device was examined in 25 participants from the healthy group. These participants completed the dexterity device test at one point in time and repeated it no more than a week later using the same testing procedure. Finally, a survey of the usability of the dexterity device was conducted with 10 participants (n = 6 healthy and n= 4 PALS), using the System Usability Questionnaire [38].

**Fig 8:**
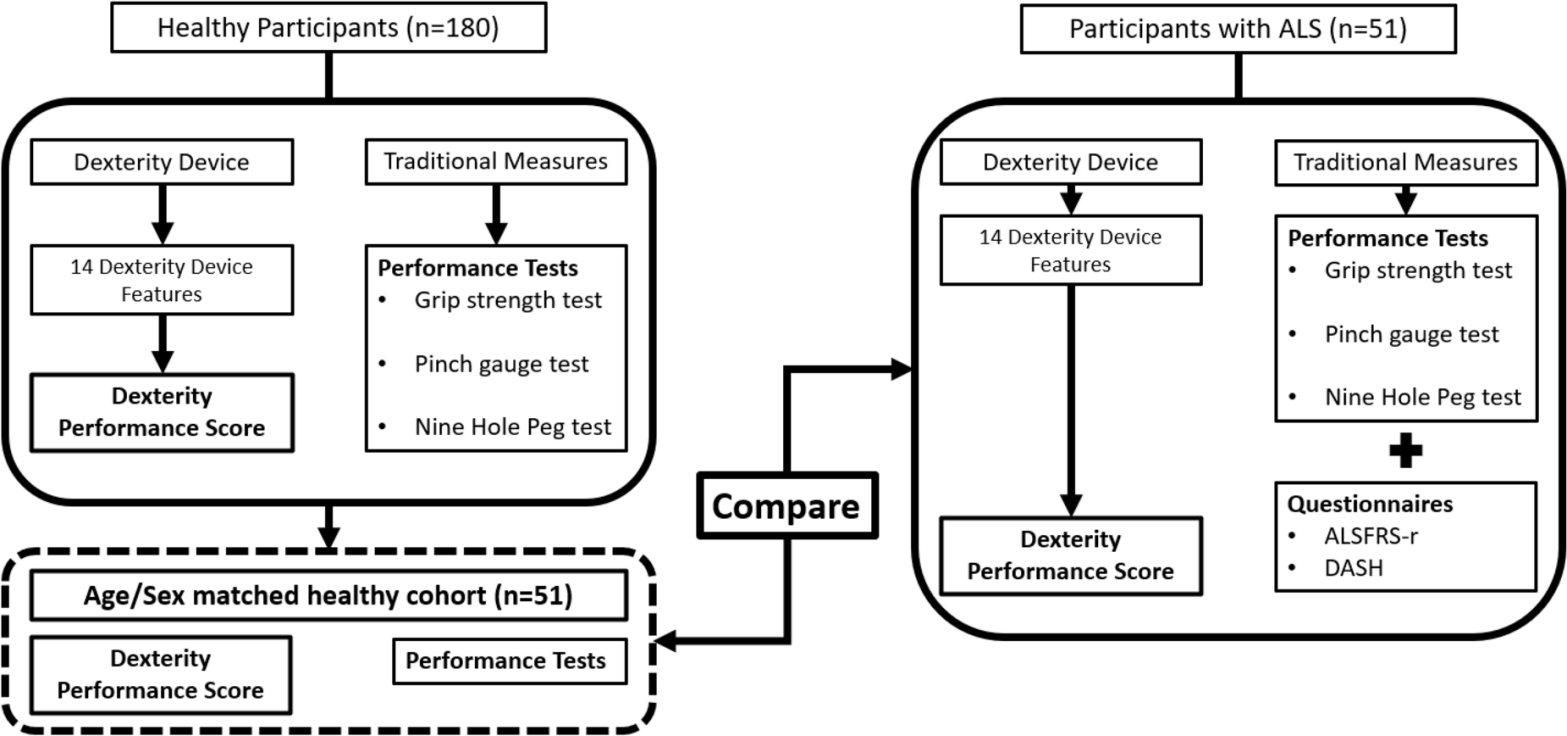
Flow chart detailing the participant testing process.

A flow chart detailing the testing procedure for the traditional tests and the dexterity device for both groups is shown in Fig 8.

## Dexterity performance score

In order to derive a dexterity performance score, the healthy participant and PALS datasets were combined into one master dataset (n = 231). The raw data underlying this study is proved in the supplementary material (S1 Data). A logistic regression model, trained using the Leave-One-Out Cross-Validation method, whereby one value was removed from the master dataset to act as the training set, was used. The logistic regression model used the maximum likelihood estimation method to find the model parameters, was fit to this data. This method calculated the probability of observing the desired outcome variables given the input data and the model. It was optimised using the likelihood function (parameters that resulted in largest sum likelihood). The model used all 14 features from the dexterity device along with the participant’s age and sex, which allowed these two effects to be accounted for in the model design. No missing data was allowed so if a participant was unable to use the dexterity device, the value for all the features were set to high values outside of a feasible performance range (i.e., 50000 seconds for time to completion or 500 millimetres or milliseconds for the hesitation features). This allowed these participants to be included in the model. A normality check was carried out and a Tukey Ladder of Powers test was carried out on all non-parametric features. If possible, all features were transformed to follow a normal gaussian distribution, however in the event a feature could not be normally transformed the transform which was closest to a normal distribution was used. This minimised the error in the model. A column indicating health status (Healthy = 1/ PALS = 0) was added to the dataset. This health status column acted as the dependent/target variable in the regression model. The output of the model was the predicted probability in log-odds units, of the event that a participant was healthy.

This logistic regression model ran sequentially the master dataset removing one participant each time, i.e., healthy participant one was removed from the master dataset, the logistic regression model was run, and the coefficients were returned for each variable in the model. These coefficients were then used to predict the continuous probability value for healthy participant one, i.e., a performance score between 0-100, p, the probability of the event that healthy participant one is healthy. This resulted in 230 distinct iterations of the logistic regression model which were used to predict a dexterity performance score for each of the removed participants. A flow chart detailing this process is shown in S5 Fig.

## Statistics

The data was analysed using MATLAB (version 2021a, The MathWorks Inc) and Microsoft Excel (version 2022). Stata (Release 17, StataCorp. 2021) and GraphPad Prism (version 9.5) were used for modelling techniques, statistical analysis and graphing purposes. Normality was assessed using the Shapiro Wilks test. The significance level for all statistical tests was set to P < 0.05 (two-sided).

The sex effect in the traditional measures and in all the dexterity device features was assessed in the healthy participant dataset using an unpaired t-test or Mann–Whitney U test for parametric and non-parametric data respectively. The age effect for all tests and features was examined using Pearson’s correlation coefficient. The age effect was assessed on three 20-year age groups (20-39, 40-59 and 60+). If a sex effect was detected for a measure or feature, then each age group was split into two groups (male and female). If no sex effect was present, the age effect was examined in the three age groups. Pairwise comparisons were carried out between these groups using a one-way ANOVA (with a Tukey’s multiple comparison correction) or Kruskal-Wallis (with a Dunn’s multiple comparison test) tests for parametric and non-parametric data respectively. A Pearson’s correlation matrix and a partial correlation, that removed the effects of age and sex was completed on the healthy participant dataset to examine these relationships.

In order to compare the test performance of the PALS to healthy performance, a subset of healthy people, age and sex matched to the PALS, was derived from the healthy cohort for analysis. These people were chosen at random with a maximum age difference of ± 3 years.

The relationship between the two questionnaires (the general DASH and the ALSFRS-rUL) on the PALS dataset were examined using a Pearson’s correlation coefficient. The ALSFRS-rUL results were divided into two score categories: normal function/mild disability (ALSFRS-rUL score: 7-12, n = 33), moderate/severe disability (ALSFRS-rUL score: 0-6, n = 18). Comparisons were carried out between these groups using a one-way ANOVA (with a Tukey’s multiple comparison correction) or Kruskal-Wallis (with a Dunn’s multiple comparison test) tests for parametric and non-parametric data respectively.

The test-retest reliability for the dexterity device was assessed with an intra-class correlation coefficient (ICC) using consistency, two-way mixed effects. As the two main outputs from the device were height and time, the reliability was assessed using a score generated by dividing the average extension height by the time to completion from the best test. The mean and standard deviation (SD) were calculated from the System Usability Scale (SUS) from a sample of ten participants.

Comparisons between the dexterity performance score and two traditional measures, grip strength and NHPT was facilitated by applying a min-max scaling transform, since none of the tests use the same scale. This was applied to the healthy dataset. The groups were split according to sex with a median value for the distribution of each metric returned. Non-parametric tests (Mann-Whitney U or Wilcoxon matched-pairs signed rank test) were carried out comparing the healthy dataset, the age/sex matched healthy people and PALS. The mean and standard deviation was used for descriptive statistics relating to the dexterity performance score. A Spearman’s correlation coefficient was used to assess the relationship between the dexterity performance scores and the various tests. A Kruskal-Wallis (with a Dunn’s multiple comparison test) was used to examine the performance scores between groups.

## Supplementary information

S1 Table Results from the performance-based assessments of hand function for healthy participants. (DOCX)

S2 Table Correlation matrices, using Pearson’s correlation coefficient, between the grip strength test and the three pinch gauge tests for healthy participants dominant and non-dominant hands. (DOCX)

S3 Table Results from the dexterity device features along with the dexterity performance score for all healthy participants (n = 180). (DOCX)

S4 Table Results from the dexterity device features along with the dexterity performance score for all healthy participants (n = 180). (DOCX)

S5 Table Results of the dexterity device features along with the dexterity performance score for PALS compared with age and sex matched healthy participants. (DOCX)

S6 Table Results of the dexterity device features along with the dexterity performance score for PALS compared with age and sex matched healthy participants. (DOCX)

S1 Fig Results for the non-dominant across three age categories for healthy participants, separated by sex. (DOCX)

S2 Fig Results of the dexterity performance score for PALS and the age/sex matched healthy subgroup. (DOCX)

S3 Fig Results (mean and 95% confidence interval) for the PALS, divided into two ALSFRS-rUL groups compared against the age/sex matched healthy cohort for dominant hand. (DOCX)

S4 Fig Images of the hand support used for the dexterity device tests. (DOCX)

S5 Fig Flowchart detailing the data cleaning steps and logistic regression model design. (DOCX)

S1 Data The raw underlying data from this study. (XLSX)
