# Supplementary material for: Clinical validation of a novel hand dexterity measurement device
Source: PLOS Digit Health. 2025 Mar 10;4(3):e0000744. doi: 10.1371/journal.pdig.0000744 (PMC11893126; doi:10.1371/journal.pdig.0000744)
Supplement: S1 Table — (DOCX) [file pdig.0000744.s001.docx]

S1 Table: Results from the performance-based assessments of hand function for healthy participants. Results are reported as mean ± standard deviation (SD).

|  | **Healthy Participants** | | | |
| --- | --- | --- | --- | --- |
| **Traditional Tests** | **Male (n=90)** | | **Female (n=90)** | |
|  | **D** | **ND** | **D** | **ND** |
| Grip Strength (kg) | 44.7 ±11.0 | 43.5 ± 11.2 | 27.1 ± 6.5 | 25.8 ± 6.1 |
| NHPT (s) | 19.9 ± 6.5 | 20.8 ± 6.7 | 17.6 ± 5.3 | 18.7 ± 2.9 |
| Tip Pinch (kg) | 7.5 ± 1.7 | 7.3 ± 1.6 | 5.6 ± 1.3 | 5.2± 1.2 |
| Palmar Pinch (kg) | 9.6 ± 1.8 | 9.2 ± 1.8 | 7.3 ± 1.4 | 6.8 ± 1.3 |
| Key Pinch (kg) | 10.4 ± 1.6 | 10.2 ± 1.9 | 7.8 ± 1.4 | 7.5 ± 1.4 |

D: Dominant; ND: Non-Dominant; n.: number; s: seconds; kg: kilogram.
