# Supplementary material for: Clinical validation of a novel hand dexterity measurement device
Source: PLOS Digit Health. 2025 Mar 10;4(3):e0000744. doi: 10.1371/journal.pdig.0000744 (PMC11893126; doi:10.1371/journal.pdig.0000744)
Supplement: S2 Table — (DOCX) [file pdig.0000744.s002.docx]

S2 Table: Correlation matrices, using Pearson’s correlation coefficient, between the grip strength test and the three pinch gauge tests for healthy participants dominant and non-dominant hands. **a** shows a standard correlation matrix for the dominant and non-dominant hand**. b** shows the partial correlation results from the same setup once the effect of age and sex has been removed.

|  | **Dominant** | | | |  | **Non-Dominant** | | | | |
| --- | --- | --- | --- | --- | --- | --- | --- | --- | --- | --- |
|  | **Correlation** | | | | | | | | | |
|  | Grip | Tip | Palmar | Key |  | Grip | Tip | Palmar | Key |  |
| Grip (kg) | 1 |  |  |  |  | 1 |  |  |  |  |
| Tip (kg) | 0.60 | 1 |  |  |  | 0.69 | 1 |  |  |  |
| Palmar (kg) | 0.76 | 0.74 | 1 |  |  | 0.79 | 0.78 | 1 |  |  |
| Key (kg) | 0.76 | 0.65 | 0.84 | 1 |  | 0.74 | 0.69 | 0.80 | 1 |  |
|  |  |  |  |  |  |  |  |  |  |  |
|  | **Correlation (Age and Sex Effect Removed)** | | | | | | | | | |
|  | Grip | Tip | Palmar | Key |  | Grip | Tip | Palmar | Key |  |
| Grip (kg) | 1 |  |  |  |  | 1 |  |  |  |  |
| Tip (kg) | 0.40 | 1 |  |  |  | 0.51 | 1 |  |  |  |
| Palmar (kg) | 0.54 | 0.63 | 1 |  |  | 0.59 | 0.66 | 1 |  |  |
| Key (kg) | 0.51 | 0.46 | 0.72 | 1 |  | 0.51 | 0.49 | 0.65 | 1 |  |
