# Supplementary material for: Clinical validation of a novel hand dexterity measurement device
Source: PLOS Digit Health. 2025 Mar 10;4(3):e0000744. doi: 10.1371/journal.pdig.0000744 (PMC11893126; doi:10.1371/journal.pdig.0000744)
Supplement: S3 Table — (DOCX) [file pdig.0000744.s003.docx]

S3 Table: Results from the dexterity device features along with the dexterity performance score for all healthy participants (n=180). The results (mean and standard deviation) are presented as sex and age groupings for the dominant hand (n =30 for all groups). Pairwise comparisons were carried out between these groups using a one-way ANOVA (with a Tukey’s multiple comparison correction) or Kruskal-Wallis (with a Dunn’s multiple comparison test) tests for parametric and non-parametric data respectively. The summary P-value between the three groups is reported.

|  | **Dominant Hand** | | | | | | | |
| --- | --- | --- | --- | --- | --- | --- | --- | --- |
|  | **M** | | | | **F** | | | |
|  | **20-39** | **40-59** | **60+** | **P** | **20-39** | **40-59** | **60+** | **P** |
| Time to Completion (s) | 4.1 ± 1.2 | 4.5 ± 1.3 | 6.6 ± 2.1 | *** | 4.4 ± 1.2 | 5.2±1.3 | 6.9±2 | *** |
| Avg. Extension Height (mm) | 117.4 ± 14.9 | 120.2 ± 18.3 | 112.4 ± 16.6 | ns | 102.2 ± 9.9 | 110.5±14.5 | 105.1±14.6 | * |
| Max Extension Height (mm) | 123.7 ± 14.2 | 128.1 ± 16.9 | 120.1 ± 16.3 | ns | 109±9.9 | 117.4±13 | 111.3±14.6 | * |
| Avg. Extension Passive Height Score | 0.9 ± 0.1 | 0.8 ± 0.1 | 0.9 ± 0.1 | ns | 0.8±0.1 | 0.9±0.1 | 0.9±0.1 | ns |
| Max Extension Passive Height Score | 0.9 ± 0.1 | 0.9 ± 0.1 | 0.9 ± 0.1 | ns | 0.9±0.1 | 0.9± 0.1 | 0.9±0.1 | ns |
| Avg. Hesitation Time (s) | 69.5 ± 48.1 | 110.4 ± 66.2 | 172.2 ± 109 | *** | 64.2±57.3 | 124.1±96.7 | 144.8±109.3 | ** |
| Avg. Hesitation Height (mm) | 1.6 ± 1.6 | 2.3 ± 2 | 3 ± 2.9 | ns | 1.5±1.6 | 2.1±2.1 | 1.74 1.51 | ns |
| Avg. No. of Hesitations per Test | 6.6 ± 3.2 | 7.2 ± 2.6 | 6 ± 2.7 | ns | 5.0±3.3 | 6.6±2.6 | 5.6±3.1 | ns |
| Avg. No. of Hesitations per Tap | 0.7±0.6 | 0.8 ± 0.6 | 1 ± 0.7 | ns | 0.6±0.6 | 0.8±0.6 | 0.7±0.6 | ns |
| Avg. Accuracy (mm) | 3.9 ± 2.4 | 4.9 ± 3.3 | 4.6 ± 2.9 | ns | 3.8±2.9 | 4.8±2.4 | 3.8±2.1 | ns |
| Decrementing amplitude (%) | 5.1 ± 5.1 | 8.4 ± 8.9 | 8 ± 7 | ns | 8.1±6.5 | 7.4±6.5 | 5.6±5 | ns |
| Avg. Extension Speed (m/s) | 0.7 ± 0.2 | 0.6 ± 0.2 | 0.5 ± 0.1 | *** | 0.5±0.1 | 0.5±0.1 | 0.4±0.1 | *** |
| Avg. Contraction Speed (m/s) | 0.6 ± 0.2 | 0.52 ± 0.18 | 0.3 ± 0.1 | *** | 0.5±0.2 | 0.4±0.1 | 0.3±0.1 | *** |
| Avg. Speed (m/s) | 0.6 ± 0.2 | 0.6 ± 0.2 | 0.4 ± 0.1 | *** | 0.5±0.1 | 0.4±0.10 | 0.3±0.1 | *** |
|  |  |  |  |  |  |  |  |  |
| **Dexterity Performance Score** | **95 ± 12** | **95 ± 7** | **70 ± 29** | ******* | **96 ± 8** | **91 ± 10** | **79 ± 20** | ******* |

P: p-value; s: seconds; mm: millimetres; %: percentage; m/s: meters per second, avg: average. Significance is denoted by (*) using the convention p < 0.05 (*), p < 0.01 (**) and p < 0.001 (***) or ns when no significance is noted.
