# Supplementary material for: Clinical validation of a novel hand dexterity measurement device
Source: PLOS Digit Health. 2025 Mar 10;4(3):e0000744. doi: 10.1371/journal.pdig.0000744 (PMC11893126; doi:10.1371/journal.pdig.0000744)
Supplement: S4 Table — (DOCX) [file pdig.0000744.s004.docx]

S4 Table: Results from the dexterity device features along with the dexterity performance score for all healthy participants (n=180). The results (mean and standard deviation) are presented as sex and age groupings for non-dominant hand (n =30 for all groups). Pairwise comparisons were carried out between these groups using a one-way ANOVA (with a Tukey’s multiple comparison correction) or Kruskal-Wallis (with a Dunn’s multiple comparison test) tests for parametric and non-parametric data respectively. The summary P-value between the three groups is reported.

|  | **Non-Dominant Hand** | | | | | | | | |
| --- | --- | --- | --- | --- | --- | --- | --- | --- | --- |
|  | **M** | | | | | **F** | | | |
|  | **20-39** | **40-59** | **60+** | **P** | **20-39** | | **40-59** | **60+** | **P** |
| Time to Completion (s) | 4.2±1 | 4.6±1.1 | 6.9±1.9 | *** | 4.6±1.4 | | 5.3±1.5 | 6.9±1.5 | *** |
| Avg. Extension Height (mm) | 112.5±12.3 | 121±11.1 | 104.8±15.2 | ** | 100.7±11.9 | | 106.7±12.1 | 103.6±10 | ns |
| Max Extension Height (mm) | 119.7±10.5 | 127.6±11.2 | 111.4±15.2 | ns | 106.8±12.6 | | 113.2±11.7 | 109.8±0.6 | ns |
| Avg. Extension Passive Height Score | 0.9±0.1 | 0.9±0.1 | 0.8±0.1 | ns | 0.9±0.1 | | 0.9±0.01 | 0.9±0.1 | ns |
| Max Extension Passive Height Score | 0.9±0.1 | 0.9±0.0 | 0.9±0.1 | ns | 0.9±0.1 | | 1±0.1 | 0.9±0.1 | ns |
| Avg. Hesitation Time (s) | 58.5±38.8 | 76.4±70.5 | 104.6±78.1 | ns | 45.7±44.2 | | 54.8±56.8 | 82.7±66.3 | ns |
| Avg. Hesitation Height (mm) | 1.2±1.7 | 1.4±1.5 | 1.8±2.4 | ns | 0.7±0.8 | | 1±1.2 | 0.9± 0.8 | ns |
| Avg. No. of Hesitations per Test | 5.3±2.4 | 5.4±3.2 | 4.7±3.3 | ns | 3.6±2.8 | | 3.9±3.2 | 4.1±3.2 | ns |
| Avg. No. of Hesitations per Tap | 0.4±0.4 | 0.6±0. | 0.6±0.6 | ns | 0.3±0.4 | | 0.4±0.5 | 0.4±0.4 | ns |
| Avg. Accuracy (mm) | 4.1±2.7 | 4.5±1.8 | 4.6±2.5 | ns | 2.6±1.8 | | 4.8±3.6 | 4.8±3.9 | * |
| Decrementing amplitude (%) | 7.8±7.2 | 5.9±4.9 | 7.2±6.1 | ns | 6.3±6.4 | | 7.6±6.5 | 7.1±5.7 | ns |
| Avg. Extension Speed (m/s) | 0.6±0.1 | 0.6±0.1 | 0.4±0.1 | *** | 0.5±0.2 | | 0.5±0.1 | 0.4±0.1 | *** |
| Avg. Contraction Speed (m/s) | 0.6±0.2 | 0.5±0.2 | 0.3±0.1 | *** | 0.5±0.2 | | 0.4±0.1 | 0.3±0.1 | *** |
| Avg. Speed (m/s) | 0.6±0.1 | 0.5±0.1 | 0.3±0.1 | *** | 0.5±0.1 | | 0.4±0.1 | 0.3±0.1 | *** |
|  |  |  |  |  |  | |  |  |  |
| **Dexterity Performance Score** | **95 ± 9** | **95 ± 7** | **68 ± 23** | ******* | **95 ± 8** | | **93 ± 12** | **79 ± 19** | ******* |

P: p-value; s: seconds; mm: millimetres; %: percentage; m/s: meters per second, avg: average. Significance is denoted by (*) using the convention p < 0.05 (*), p < 0.01 (**) and p < 0.001 (***) or ns when no significance is noted.
