# Supplementary material for: Clinical validation of a novel hand dexterity measurement device
Source: PLOS Digit Health. 2025 Mar 10;4(3):e0000744. doi: 10.1371/journal.pdig.0000744 (PMC11893126; doi:10.1371/journal.pdig.0000744)
Supplement: S5 Table — (DOCX) [file pdig.0000744.s005.docx]

S5 Table: Results of the dexterity device features along with the dexterity performance score for PALS compared with age and sex matched healthy participants. The results (means and standard deviation) are presented for the dominant hand separated by sex. Paired t-tests or Wilcoxon matched-pairs signed rank tests were carried out between the groups.

|  | **Dominant** | | | | | | | |
| --- | --- | --- | --- | --- | --- | --- | --- | --- |
|  | **Male (N = 32)** | | | | **Female (N = 19)** | | | |
|  | **Healthy** | **PALS** | **P** | **Healthy** | | **PALS** | **P** |  |
| Time to Completion (s) | 5.9±2.3 | 10.9 ± 8.4 | *** | 6±1.5 | | 14.5 ± 13.6 | *** |  |
| Avg. Extension Height (mm) | 113±19.1 | 87.4±38.5 | *** | 105.6±15.8 | | 74.3±38.1 | ** |  |
| Max Extension Height (mm) | 121.9±17.9 | 95.7±39.1 | *** | 111.1±15.7 | | 81.5±39.9 | * |  |
| Avg. Extension Passive Height Score | 0.8± 0.1 | 0.7 ± 0.3 | * | 0.9±0.1 | | 0.6 ± 0.3 | ** |  |
| Max Extension Passive Height Score | 0.9± 0.1 | 0.8 ± 0.3 | ns | 0.9±0.1 | | 0.7 ± 0.3 | ** |  |
| Avg. Hesitation Time (s) | 0.2± 0.1 | 0.3 ± 0.9 | * | 0.1± 0.1 | | 0.6 ± 1.6 | ns |  |
| Avg. Hesitation Height (mm) | 3.1± 2.6 | 2.7 ± 8.7 | ** | 2.1±2 | | 6.9 ± 15.7 | ns |  |
| Avg. No. of Hesitations per Test | 7.3±2.4 | 5.3 ± 8.9 | ** | 6.4±2.8 | | 8.6 ± 14.9 | ns |  |
| Avg. No. of Hesitations per Tap | 1.1± 0.6 | 2.0 ± 8.8 | ** | 0.7±0.7 | | 5.6 ± 15.7 | ns |  |
| Avg. Accuracy (mm) | 5.1± 3 | 20.8 ± 87.6 | ns | 4.3±2.5 | | 56.1±156.5 | ns |  |
| Decrementing amplitude (%) | 9.7±8.2 | 22.7 ± 88.2 | ns | 5.7±4.2 | | 68.6±154.2 | ns |  |
| Avg. Extension Speed (m/s) | 0.5±0.2 | 0.2 ± 0.2 | *** | 0.4±0.1 | | 0.23 ± 0.2 | ** |  |
| Avg. Contraction Speed (m/s) | 0.4±0.2 | 0.2 ± 0.2 | ** | 0.4± 0.1 | | 02. ± 0.1 | ** |  |
| Avg. Speed (m/s) | 0.4± 0.1 | 0.2 ± 0.2 | *** | 0.4±0.1 | | 0.2 ± 0.1 | *** |  |
|  |  |  |  |  | |  |  |  |
| **Dexterity Score** | **75 ± 27** | **37 ± 32** | ******* | **87 ± 12** | | **53 ± 38** | ****** |  |

P: p-value; s: seconds; mm: millimetres; %: percentage; m/s: meters per second, avg: average. Significance is denoted by (*) using the convention p < 0.05 (*), p < 0.01 (**) and p < 0.001 (***) or ns when no significance is noted.
