# Supplementary material for: Clinical validation of a novel hand dexterity measurement device
Source: PLOS Digit Health. 2025 Mar 10;4(3):e0000744. doi: 10.1371/journal.pdig.0000744 (PMC11893126; doi:10.1371/journal.pdig.0000744)
Supplement: S1 Fig — (DOCX) [file pdig.0000744.s007.docx]

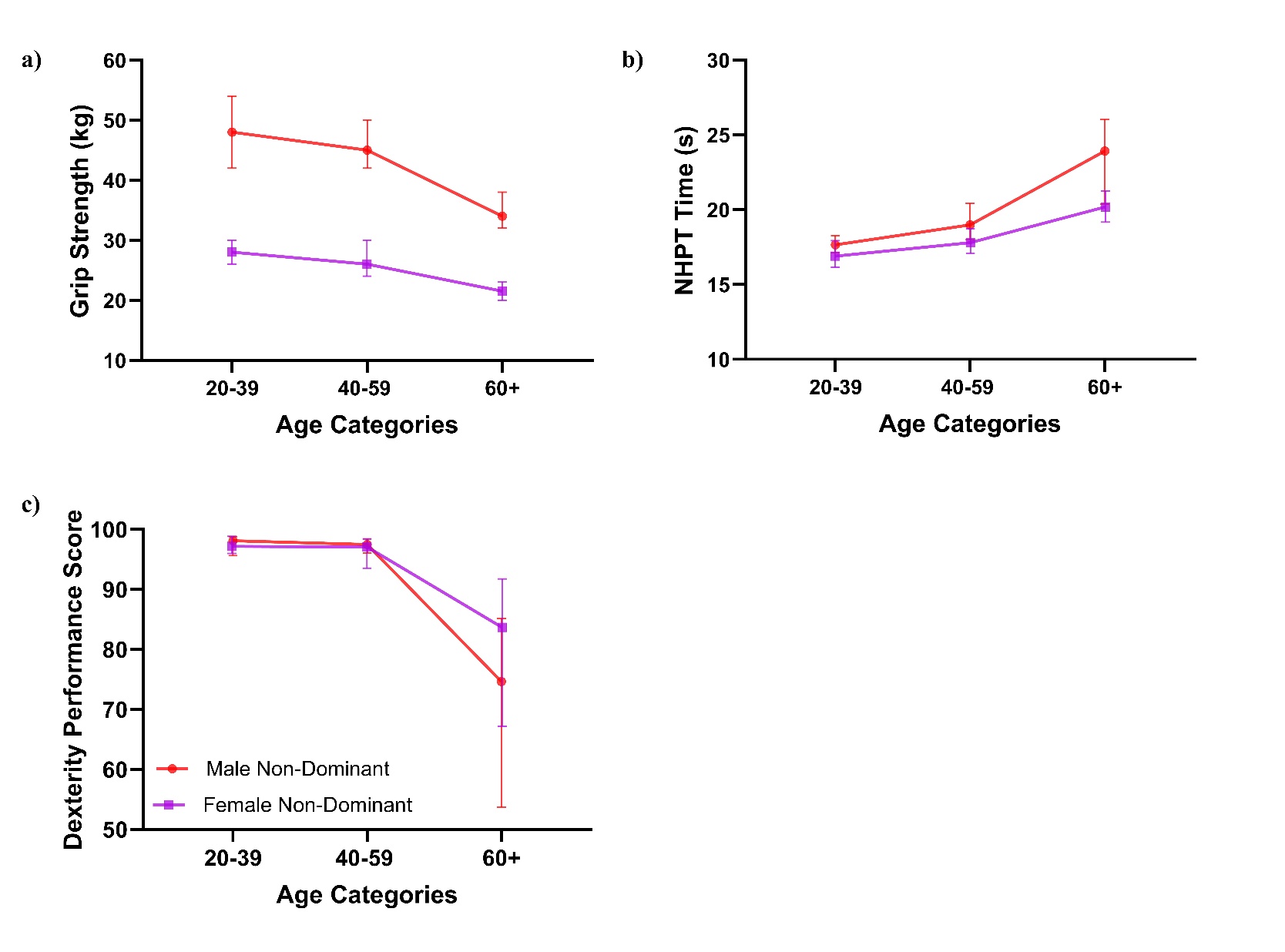


S1 Figure: Results for the non-dominant across three age categories for healthy participants, separated by sex. **a** The grip strength test. **b** The nine hole peg test (NHPT). **c** The dexterity performance score (from 50 units). The median and 95% confidence intervals are plotted and separated by sex and hand dominance.
