# Supplementary material for: Clinical validation of a novel hand dexterity measurement device
Source: PLOS Digit Health. 2025 Mar 10;4(3):e0000744. doi: 10.1371/journal.pdig.0000744 (PMC11893126; doi:10.1371/journal.pdig.0000744)
Supplement: S2 Fig — (DOCX) [file pdig.0000744.s008.docx]

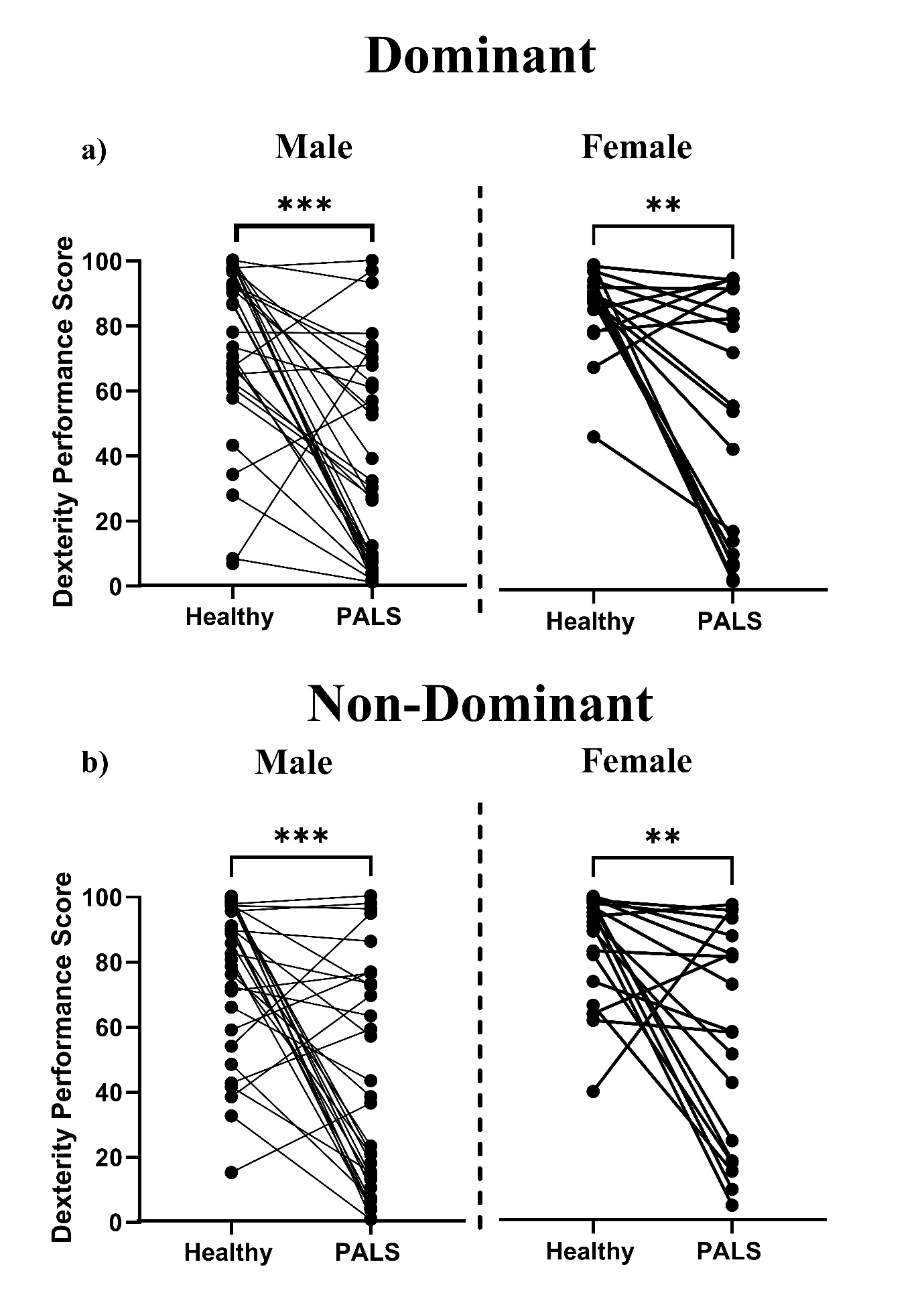


S2 Figure: Results of the dexterity performance score for PALS and the age/sex matched healthy subgroup. **a** shows the results (mean and standard deviation) for the dominant with **b** highlighting the non-dominant hand. Significance is denoted by (*) using the convention p < 0.05 (*), p < 0.01 (**) and p < 0.001 (***) or ns when no significance is noted. A Wilcoxon matched pairs signed rank test was performed for comparisons.
