# Supplementary material for: Clinical validation of a novel hand dexterity measurement device
Source: PLOS Digit Health. 2025 Mar 10;4(3):e0000744. doi: 10.1371/journal.pdig.0000744 (PMC11893126; doi:10.1371/journal.pdig.0000744)
Supplement: S3 Fig — (DOCX) [file pdig.0000744.s009.docx]

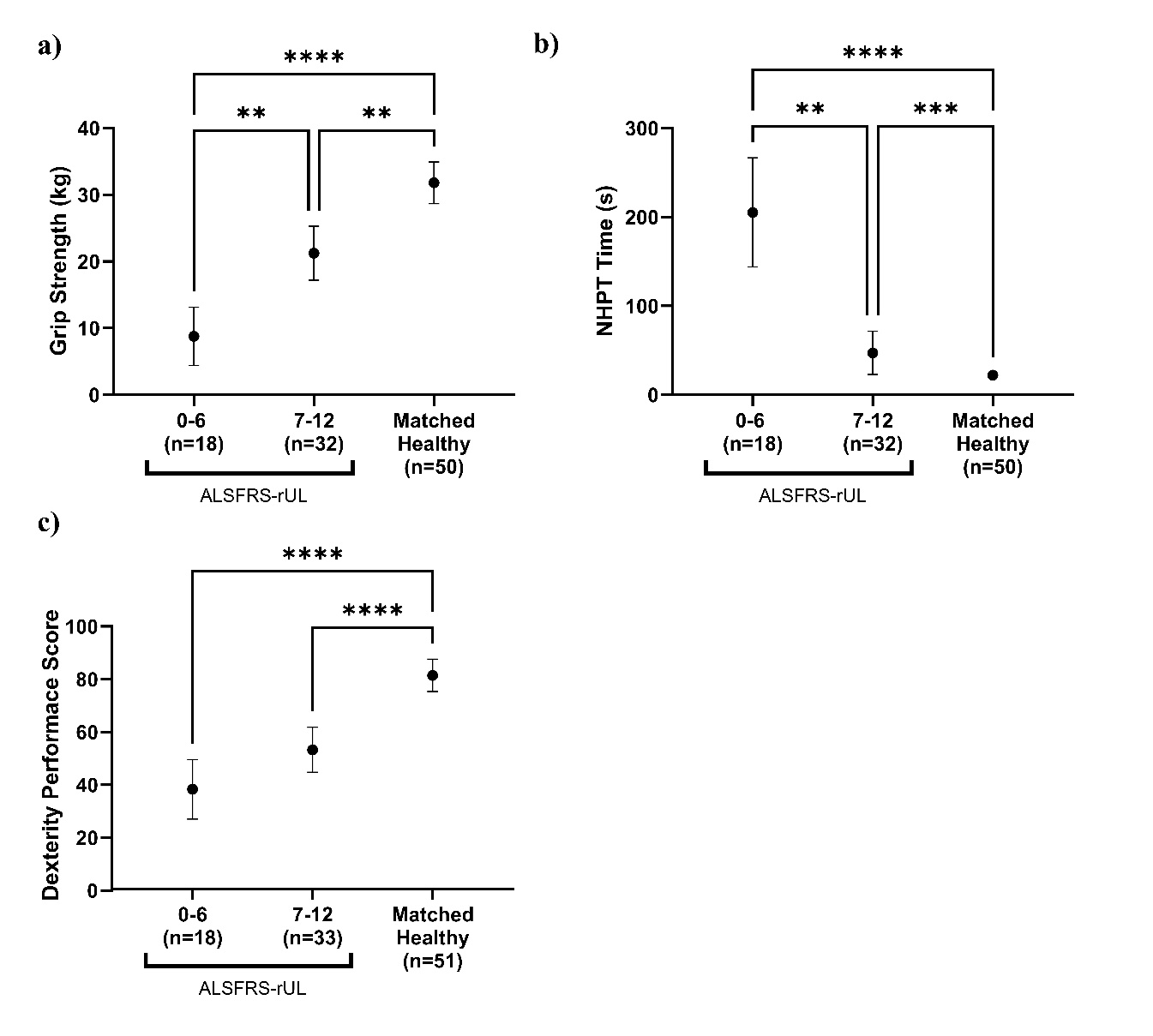


S3 Figure: Results (mean and 95% confidence interval) for the PALS, divided into two ALSFRS-rUL groups compared against the age/sex matched healthy cohort for dominant hand. **a** Grip strength test. **b** Nine hole peg test (NHPT). **c** Dexterity performance score. Pairwise comparisons were carried out using a Kruskal-Wallis (with a Dunn’s multiple comparison test) tests. Significance is denoted by (*) using the convention p < 0.05 (*), p < 0.01 (**), p < 0.001 (***) or p <0.0001 (****) or ns when no significance is noted.
