# Supplementary material for: Clinical validation of a novel hand dexterity measurement device
Source: PLOS Digit Health. 2025 Mar 10;4(3):e0000744. doi: 10.1371/journal.pdig.0000744 (PMC11893126; doi:10.1371/journal.pdig.0000744)
Supplement: S4 Fig — (DOCX) [file pdig.0000744.s010.docx]

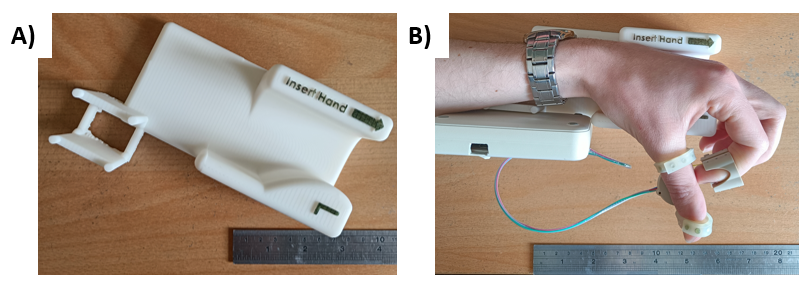


S4 Figure: Images of the hand support used for the dexterity device tests. **a** The custom hand support to standardised orientation is shown on its own and **b** during a test procedure.
